# Overlapping filter bank convolutional neural network for multisubject multicategory motor imagery brain-computer interface

**DOI:** 10.1186/s13040-023-00336-y

**Published:** 2023-07-11

**Authors:** Jing Luo, Jundong Li, Qi Mao, Zhenghao Shi, Haiqin Liu, Xiaoyong Ren, Xinhong Hei

**Affiliations:** 1 Shaanxi Key Laboratory for Network Computing and Security Technology, School of Computer Science and Engineering, Xi’an University of Technology, Xi’an, Shaanxi People’s Republic of China; 2 Human-Machine Integration Intelligent Robot Shaanxi University Engineering Research Center, Xi’an University of Technology, Xi’an, Shaanxi People’s Republic of China; 3 Department of Otolaryngology Head and Neck Surgery & Center of Sleep Medicine, The Second Affiliated Hospital of Xi’an Jiaotong University, Xi’an, Shaanxi People’s Republic of China

**Keywords:** Brain-computer interface (BCI), Multisubject BCI, Motor imagery (MI), Convolutional neural network (CNN), Overlapping filter bank

## Abstract

**Background:**

Motor imagery brain-computer interfaces (BCIs) is a classic and potential BCI technology achieving brain computer integration. In motor imagery BCI, the operational frequency band of the EEG greatly affects the performance of motor imagery EEG recognition model. However, as most algorithms used a broad frequency band, the discrimination from multiple sub-bands were not fully utilized. Thus, using convolutional neural network (CNNs) to extract discriminative features from EEG signals of different frequency components is a promising method in multisubject EEG recognition.

**Methods:**

This paper presents a novel overlapping filter bank CNN to incorporate discriminative information from multiple frequency components in multisubject motor imagery recognition. Specifically, two overlapping filter banks with fixed low-cut frequency or sliding low-cut frequency are employed to obtain multiple frequency component representations of EEG signals. Then, multiple CNN models are trained separately. Finally, the output probabilities of multiple CNN models are integrated to determine the predicted EEG label.

**Results:**

Experiments were conducted based on four popular CNN backbone models and three public datasets. And the results showed that the overlapping filter bank CNN was efficient and universal in improving multisubject motor imagery BCI performance. Specifically, compared with the original backbone model, the proposed method can improve the average accuracy by 3.69 percentage points, F1 score by 0.04, and AUC by 0.03. In addition, the proposed method performed best among the comparison with the state-of-the-art methods.

**Conclusion:**

The proposed overlapping filter bank CNN framework with fixed low-cut frequency is an efficient and universal method to improve the performance of multisubject motor imagery BCI.

## Background

Brain computer interface (BCI) is a communication interface directly established between the brain and the computer, which can realize the interconnection between brain and external objects, and achieve the interactive integration of biological intelligence and machine intelligence. It has important applications in the fields of medicine, neurobiology and psychology [1]. Many mature paradigms have emerged in the BCI field, including motor imagery BCI, steady-state visual evoked potential (SSVEP) BCI, P300 visual-evoked potentials BCI and emotional BCI [1, 2].

Motor imagery electroencephalography (MI-EEG) is a kind of endogenous spontaneous EEG with low environmental requirements. Thus, MI-EEG is widely used in BCI. The MI-BCI system collects EEG signals when the subject performs specific motor imagery, then recognizes the MI content according to the EEG signals, and converts the recognition results into control commands of the peripheral devices [3]. EEG signals have the characteristics of a low signal-to-noise ratio and low spatial resolution, so effective features and classifiers are the keys to the success of the MI recognition system, and BCI researchers have increasingly proposed many algorithms for MI classification.

### Common spatial pattern-related method

The common spatial pattern (CSP) algorithm and its variants have been widely applied to construct spatial filters and extract highly discriminative features in EEG-based MI classification by maximizing the variance difference between two classes of EEG signals [4]. The outstanding performance of the filter bank common spatial pattern (FBCSP) won the BCI Competition IV in the 2a dataset and 2b dataset [5]. The FBCSP used a filter bank consisting of 9 nonoverlapping subband bandpass filters covering the frequency range of 4 to 40 Hz to preprocess the signal. Then, the CSP features were extracted and selected for specific subjects by the mutual information-based rough set reduction algorithm and fed to the naïve Bayesian Parzen window classifier. The filter bank regularized common spatial pattern was proposed to simultaneously solve the dependency problems on frequency bands and sample-based covariance estimation, and the proposed method improved the mean classification accuracy compared with other CSP-based methods [6]. Zhang proposed a hybrid network consisting of a CNN and a long-term short-term memory network for extracting temporal and spatial features from CSP features [7].

### Deep learning-based method

Recently, deep learning algorithms have developed quickly, and related algorithms have been proposed for EEG-based BCI. In particular, CNNs have been widely used in EEG-based MI classification due to their ability to effectively extract temporal and spatial features from EEG signals. Schirrmeister proposed a Shallow ConvNet and a Deep ConvNet for end-to-end MI-based EEG recognition and showed better performance compared with the FBCSP algorithm. In addition, the CNN visualization results showed that the proposed model learned to use spectral power characteristics from different frequency bands [8]. EEGNet was proposed by Lawhern to suggest that a compact CNN can be applied and provide robust performance across many BCI paradigms, such as P300 event-related potential, feedback error-related negativity, movement-related cortical potential and sensory motor rhythm (MI recognition) [9]. Chen designed a deep learning approach termed filter bank spatial filtering and temporal-spatial CNN for MI decoding. Filter bank spatial filtering extracts the feature presentation of raw EEG signals, and the temporal-spatial CNN implements a decoding procedure. A stagewise training strategy including optimizing the triplet loss and cross-entropy loss was proposed to mitigate the optimization difficulty [10]. Li proposed an end-to-end EEG decoding framework that regards the original multichannel EEG as the input and improves the classification accuracy through a channel projection mixed-scale convolutional neural network (CP-MixedNet) and amplitude perturbation data augmentation [11]. Sakhavi proposed a novel filter bank convolutional network (FBCNet) for MI classification, which extracted a multiview data representation through a filter bank, extracted spatial features through depthwise convolutional layers, and effectively aggregated temporal information by the proposed variance layer [12]. Zhao built a multibranch 3D convolutional neural network, where the 3D representation was generated by transforming EEG signals into a series of 2D arrays focusing on the spatial distribution of channels [13]. Li proposed a novel temporal-spectral-based squeeze-and-excitation feature fusion network, which extracted high-dimensional temporal features and discriminative spectral representations from raw EEG signals via deep-temporal convolution block and multilevel wavelet convolutions, respectively. Channelwise discriminative responses were highlighted by constructing interdependencies among different domain features [14]. Five adaptive schemes of the EEG-BCI system based on CNN were proposed for decoding MI-EEG. The adaptive transfer learning method fine-tuned an extensively trained, pretrained model and adjusted it to adapt the target subject [15].

### Multisubject calibration-free MI-BCI

At present, research on MI-BCIs mainly focuses on subject-dependent systems, in which a model is built for a single target subject and has achieved satisfactory results [6, 8]. However, a subject-dependent system needs to collect data for calibrating the target subject, which is time-consuming and only applicable to the target subject. Therefore, research on multisubject calibration-free BCI systems has appeared. Kwon constructed a large MI-based EEG database consisting of 54 subjects performing left- and right-hand MI and proposed a subject-independent multibranch CNN framework for subject-independent BCI. The spectral–spatial inputs are individually trained through the CNN and then combined by a concatenation fusion technique to make predictions [16]. Zhang proposed a convolutional recurrent attention model for subject-independent EEG signal analysis. Specifically, they split an EEG trial into multiple temporal slices, utilized the spatial-temporal block to extract the spatial-temporal information of every temporal slice, and finally, leveraged a recurrent attention mechanism to explore the temporal dynamics among different temporal slices. The improved performance in the experiment indicated that the proposed convolutional recurrent attention model can utilize potential invariant EEG patterns among different subjects [17]. To improve the classification accuracy of multisubject motor imagery, Autthasan designed a novel end-to-end multitask learning architecture called MIN2Net, which applied a deep metric learning method in a multitask autoencoder model to learn discriminative potential representations and make predictions simultaneously [18]. Luo proposed a twin cascaded softmax convolutional neural network (TCSCNN) for multisubject MI-BCIs. The cascaded softmax structure was applied to achieve subject recognition and MI recognition simultaneously, and the twin EEG and twin structure were employed to further improve the performance [19].

In multisubject MI-BCI systems, the individual differences in EEG signals cause great difficulties for research [20]. In particular, the effective frequency band related to event-related desynchronization (ERD) and event-related synchronization (ERS) varies from subject to subject. Utilizing the discriminative information of different frequency bands is key to improving the capability of multisubject MI-BCI, but existing CNN models perform poorly in this aspect. This paper presents a novel overlapping filter bank CNN framework for multisubject motor imagery EEG recognition. Through the proposed overlapping filter bank, the filtered EEG forces the CNN to learn from different frequency bands. To combine the discriminative ability from different frequency bands, ensemble probability from multiple CNNs is employed to make predictions.

The main contributions of this paper are as follows.

A) We develop the overlapping filter bank CNN framework, which is universal and effective for different CNN backbones, to capture discriminative information from multiple EEG frequency bands for multisubject MI-BCI.
B) Compared with the traditional nonoverlapping filter bank with narrow pass bands, the proposed novel overlapping filter bank with broad pass bands and fixed low-cut frequency is more suitable for CNN based multisubject MI recognition.
C) Comprehensive experimental evaluations on three benchmark datasets are applied to demonstrate the effectiveness and universality of the proposed overlapping filter bank CNN framework.

The rest of this paper is organized as follows. Methods section presents the proposed overlapping filter bank CNN framework. Experimental data section introduces the dataset and experimental settings in detail. Then, the experimental results and discussion are presented in Results and discussion section. Conclusion section gives the conclusions.

## Methods

### Analysis of FBCSP

Using discriminative information from multiple frequency bands is a promising method to improve the MI classification performance. The FBCSP algorithm is a successful example of using discriminative information from multiple frequency bands and won the champion of BCI Competition IV dataset 2a and dataset 2b [5]. To date, the FBCSP has been applied and modified by numerous researchers [4, 6, 21].

In the FBCSP algorithm, a nonoverlapping bandpass filter bank is employed first to decompose the EEG into multiple frequency bands. Thus, the discriminative information of different frequency bands can be collected in the following feature extraction. Second, the CSP algorithm is applied to design a spatial filter, and the log variance feature is extracted from the EEG signals filtered by the spatial filter.

1 $${v}_{p}=log\frac{var({W}_{p}E)}{{\sum }_{i=1}^{I}var({W}_{i}E)}$$

where *E* is the raw EEG signals and *W*_*i*_ is the *i* th spatial filter [22]. As CSP is a supervised feature learning method using a spatial filter to maximize the variance differences between the two classes of EEG signals, it is more like a classification method rather than a feature extraction method. The CSP features can directly be used in classification by a threshold. However, the discriminative ability of a single classifier built on a specific EEG frequency band is usually weak in classification. Thus, the following feature selection and classifier training procedures are applied.

Analysis of the FBCSP method shows that it is indeed an ensemble learning method. Multiple weak classifiers (CSP features extracted in different frequency bands) are combined (training of classifier) to build a strong classifier. This is a valuable experience that should be used in CNN-based MI recognition methods.

However, this framework of training and combining weak classifiers from different frequency bands is difficult to transfer directly. Compared with the simple linear spatial filter provided by the CSP, the complexity of the CNN model is much higher. If the input bandpass filtered EEG does not have enough discriminative information, the CNN begins to search for less discriminative information and even indiscriminative information, which finally results in overfitting. In conclusion, the CNN model performs better based on wide frequency band EEG, which contains more discriminative information. The discriminative information in the EEG signals filtered by a narrow bandpass filter (4 Hz in FBCSP) is insufficient, so a narrow bandpass filter easily causes overfitting in the CNN-based method.

### Overlapping filter bank

To supply enough discriminative information for the CNN model, the filter bank should have a wider passband. Compared with the nonoverlapping filter bank applied in FBCSP, filter banks with overlapping frequency bands are applied before the CNN model in this paper. Two kinds of overlapping filter banks are proposed. The first type is a fixed-low-cut-frequency filter bank, the second type is a sliding-low-cut-frequency filter bank, and the specific setting is described in Table 1.

**Table 1:** The bandpass filters included different overlapping filter banks

Filter bank	First filter	Second filter	Third filter		Last filter
Fixed-0 Hz	0–8 Hz	0–12 Hz	0–16 Hz	…	0–36 Hz
Fixed-4 Hz	4–12 Hz	4–16 Hz	4–20 Hz	…	4–36 Hz
Sliding-0 Hz	0–12 Hz	4–16 Hz	8–20 Hz	…	24–36 Hz
Sliding-4 Hz	4–16 Hz	8–20 Hz	12–24 Hz	…	24–36 Hz

### Overlapping filter bank CNN

**Fig. 1:**
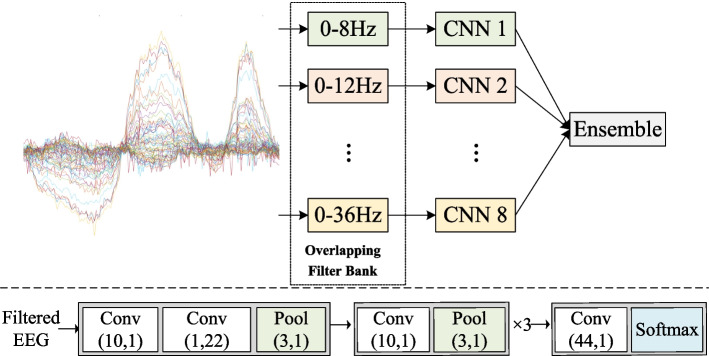
Illustration of the overlapping filter bank CNN and the structure of the CNN

Due to the powerful feature learning capabilities of the CNN model, it is applied to extract discriminative information from multiple EEG frequency bands. The flowchart of the proposed overlapping filter bank CNN (OFBCNN) is shown in the upper part of the Fig. 1. As the proposed framework is universal for different CNN backbones, the structure of Deep ConvNet [8] is shown in the lower part of the Fig. 1 as an example of the CNN. Multiple CNN models are trained by the EEG signal filtered by the overlapping filter bank, and the output probabilities are combined to make the final prediction in the testing stage. The details are given below.

First, the raw EEG is filtered by a bandpass filter:

2 $$\widehat{{E}_{n}}={F}_{n}(E)$$

where *E* and $\widehat{{E}_{n}}$ represents the raw EEG and the filtered EEG, ${F}_{n}$ represents the *n*th bandpass filter in the filter bank. For example, if the overlapping filter bank with 0 Hz fixed-low-cut-frequency (as shown in Table 1) is applied, the passband of first bandpass filter is 0–8 Hz, and the passband of second bandpass filter is 0–12 Hz, and so on. Second, the EEG signals filtered by same bandpass filter and the related motor imagery labels are used to train each CNN backbone model using cross entropy loss. Finally, we obtain multiply CNN models corresponding to multiply frequency bands.

In the testing stage, the prediction probability of each frequency band is the output of the corresponding CNN model:

3 $${O}_{n}={M}_{n}(\widehat{{E}_{n}})$$

where ${M}_{n}$ is the CNN model for $\widehat{{E}_{n}}$ and ${O}_{n}$ is the output prediction probability of ${M}_{n}$. The output probabilities of all CNN models are summed to determine the final prediction:

4 $$L=\mathrm{argmax}(\sum_{n=1}^{N}{O}_{n})$$

where *N* is the total number of bandpass filters in the overlapping filter bank and *L* is the predicted label.

### CNN backbone

Four popular CNN backbone models, including shallow ConvNet, deep ConvNet [8], EEGNet [9] and ATCNet [23], are applied in the proposed overlapping filter bank CNN framework (OFBCNN) in this paper.

Deep ConvNet is a deep CNN proposed by Schirrmeister that includes four “Conv Pool Blocks” and a classification layer. A standard “Conv Pool Block” includes a convolutional layer and a max pooling layer. However, the first “Conv Pool Block” includes a temporal convolution to extract low-level temporal feature representations, a spatial filter (convolution along all EEG channels) and a max pooling layer. For specific network details, see [8].

Shallow ConvNet is a shallower network with only one “Conv Pool Block”, including a temporal convolution, spatial filtering layer and an average pooling layer. This architecture is inspired by the FBCSP and uses the first two convolutional layers to replace the bandpass and CSP spatial filters in the FBCSP. For specific network details, see [8].

EEGNet is a robust end-to-end network showing outstanding performance in multiple BCI paradigms, i.e., P300 visual evoked potential, error-related negative response (ERN), motor-related cortical potential (MRCP) and sensorimotor rhythm (SMR). EEGNet consists of three convolutional pooling blocks and a softmax layer. Each convolutional layer is followed by the batch norm, ELU activation function, max pooling and dropout. For specific details of the network, see [9].

ATCNet [23] is an attention-based temporal convolutional network, which mainly consists of convolutional block, multi-head attention block, and temporal convolutional block. The raw EEG is input into the convolutional block, and the output high-level temporal features of temporal convolution block are input into a fully connected layer with a softmax classifier to make prediction. For specific details of the network, see [23].

## Experimental data

### BCI Competition IV dataset 2a

The BCI Competition IV dataset 2a includes a four-category MI-based EEG signal recognition task. This dataset includes MI-based EEG signals from 9 subjects. For each subject, there were 72 trials in each class in the training set and test set, recorded at different times. According to four types of prompts, including MI of the left hand, right hand, tongue, and foot, the subjects performed MI, and EEG signals were monitored from 22 channels. The sampling rate of the EEG signals was 250 Hz, and the resolution of the amplifier was 100 mV. In each trial, the prompt appeared on the screen in seconds, and the execution time of the MI task was between the third second and sixth second. A 0.5–100 Hz bandpass filter and a 50 Hz notch filter were used to preprocess the collected EEG signals. After recording, an expert marked the artefact trial. For more details, see [24]. The experiments in this paper used all the training data of 9 subjects as the training set and all the test data of 9 subjects as the test set to construct a multisubject MI recognition task.

### High-Gamma dataset

The High-Gamma (HG) dataset is a large-scale EEG dataset composed of four types of EEG signals from 14 subjects performing motor execution tasks (left hand, right hand, feet, and rest) [8]. For each subject, there were 13 runs and approximately 1,000 four-second trials. The first 11 runs included approximately 880 trials belonging to the training set, and the last 2 runs consisted of approximately 160 trials belonging to the test set. EEG signals were collected from 128 channels (44 channels were used in the following experiments according to Braindecode [8]) and resampled at 250 Hz. More details can be found in [8]. All training EEG signals from 14 subjects were used as the training set, and all the testing EEG signals from 14 subjects were used as the test set for multisubject EEG recognition.

### OpenBMI dataset

The OpenBMI (BMI) dataset consisting EEG signals from 54 subjects was collected by Korea University [25]. In BMI dataset, each subject executed two EEG signal collection sessions on different days. The data in each session included a train set and a test set, where each set contains 100 trials (50 left hand motor imagery tasks and 50 right hand motor imagery tasks). EEG signals were collected from 64 channels and sampled at 1000 Hz (We resampled it at 250 Hz). In the EEG collection, the first 3 s of each trial began with a black fixation cross that appeared at the center of the monitor to prepare subjects for the MI task. Afterwards, the subject performed the imagery task of grasping with the appropriate hand for 4 s when the right or left arrow appeared as a visual cue. More details can be found in [25]. In our experiments with OpenBMI dataset, we removed 33 MI blind subjects, so EEG signals from 21 subjects were employed (subject 1, subject 2, subject 3, subject 5, subject 6, subject 9, subject 17, subject 18, subject 19, subject 21, subject 22, subject 28, subject 29, subject 32, subject 33, subject 36, subject 37, subject 43, subject 44, subject 45, subject 52).

### Experimental setup

The EEG signals were only minimally preprocessed to encourage the CNN model to learn features by itself. The 4.5 s EEG segment was used in dataset 2a and HG dataset, from 0.5 s before the MI prompt appeared to 4 s after it appeared. The all 4 s EEG trial was used in OpenBMI dataset, from the MI prompt appeared to 4 s after it appeared. The EEG signals were filtered by the overlapping filter bank, and then the filtered EEG signals were normalized using channel exponential running standardization, as configured in [8]. After that, the EEG signals were input into each CNN model. The Adam optimization method was used in the training phase [26].

In the experiment, a sever with Intel Core i7-10700, NVIDIA GeForce RTX 3090 and 48 GB RAM was applied. And the proposed framework was implemented in Python using the PyTorch [27] and Braindecode [8] frameworks.

To reduce the influence of randomness and epoch number, the average test accuracy, F1 score and AUC were used to evaluate the accuracy of the model. In addition, the variance in the test accuracy was used to assess the convergence of the model.

## Results and discussion

To verify the effectiveness, universality and feasibility of the proposed overlapping filter bank CNN algorithm, a series of experiments were conducted. First, OFBCNN (including sliding or fixed Low-Cut Frequency) was compared with the original CNN and the CNN with the nonoverlapping filter bank in the multisubject MI recognition task. Second, the performance of OFBCNN was compared with the state-of-the-art algorithm. Finally, a parameter sensitivity test was performed.

### Performance comparison of the proposed OFBCNN with the original CNN and the nonoverlapping filter bank CNN in multisubject MI recognition

The performance of OFBCNN (including sliding or fixed Low-Cut Frequency) was compared with the original backbone CNN without the overlapping filter bank and the CNN with the nonoverlapping filter bank in the multisubject MI recognition task. In dataset 2a and dataset HG, the maximum number of epochs for training was set as 500 to ensure the convergence of the model. Because of the larger sample number in OpenBMI dataset, the maximum number of epochs for training was set as 300. The average testing accuracy, F1 score and AUC of last 100 epoch were applied in the experimental evaluation.

The results (measured by accuracy/F1 Score/AUC) are given in Table 2. The first column represents the original CNN model name, dataset, and low-cut frequency of the filter bank encoded in the form of a “model-dataset-filter”. For example, “EEGNet-2a-0” indicates that OFBCNN was built based on the EEGNet backbone model, evaluated on the BCI Competition IV dataset 2a, and the low-cut frequency of the filter bank is 0 Hz. NOFBCNN denotes the nonoverlapping filter bank CNN with bandpass filters including 0–4 Hz (if the low-cut frequency is 0), 4–8 Hz, 8–12 Hz, 12–16 Hz … 32–36 Hz (as the configuration in FBCSP). Due to the existing of the significant low frequency noise in dataset BMI, the low-cut frequency of the filter bank is set as 4 Hz. The best measurements of each experimental setting are shown in bold.

**Table 2:** Performance comparison of the OFBCNN. The results are measured by accuracy (%)/F1 score/AUC

	Original	NOFBCNN	Sliding OFBCNN	Fixed OFBCNN
EEGNet-2a-0	68.57/0.66/0.89	60.76/0.58/0.87	65.15/0.64/0.89	**71.76**/**0.71**/**0.91**
Shallow-2a-0	72.80/0.74/**0.93**	68.31/0.68/0.88	71.51/0.71/0.91	**76.57**/**0.76**/**0.93**
Deep-2a-0	71.42/0.72/0.91	65.14/0.64/0.87	68.27/0.67/0.89	**75.53**/**0.75**/**0.93**
ATCNet-2a-0	76.96/0.76/0.92	73.14/0.72/0.91	73.41/0.73/0.91	**79.63**/**0.79**/**0.95**
EEGNet-2a-4	61.68/0.58/0.84	54.49/0.52/0.82	59.23/0.57/0.85	**63.40**/**0.62**/**0.88**
Shallow-2a-4	65.44/0.67/0.87	62.58/0.62/0.84	67.65/0.67/0.88	**69.97**/**0.69**/**0.90**
Deep-2a-4	56.76/0.57/0.82	58.26/0.56/0.81	**61.89**/**0.61**/**0.85**	61.38/0.60/**0.85**
ATCNet-2a-4	71.75/0.71/0.89	66.97/0.66/0.87	68.40/0.68/0.88	**76.19**/**0.76**/**0.93**
EEGNet-HG-0	83.16/0.83/**0.97**	70.08/0.67/0.95	76.20/0.75/0.96	**85.60**/**0.85**/**0.97**
Shallow-HG-0	88.69/0.88/0.97	87.50/0.87/0.97	89.19/**0.89/0.98**	**89.91**/**0.89/**0.97
Deep-HG-0	89.68/0.89/0.98	80.88/0.80/0.96	83.94/0.83/0.97	**91.90**/**0.91**/**0.99**
ATCNet-HG-0	92.00/0.92/0.97	91.96/0.91/0.98	91.65/0.91/0.98	**93.25**/**0.93**/**0.99**
EEGNet-HG-4	79.11/0.72/0.95	64.85/0.62/0.93	71.29/0.70/0.94	**80.18**/**0.79**/**0.96**
Shallow-HG-4	87.15/0.86/0.97	84.38/0.84/0.96	86.76/0.80/**0.98**	**87.51**/**0.87**/**0.98**
Deep-HG-4	80.37/0.78/0.95	75.85/0.75/0.95	80.11/0.79/0.96	**82.16**/**0.82**/**0.97**
ATCNet-HG-4	88.03/0.88/0.96	89.24/0.89/**0.98**	89.19/0.89/**0.98**	**91.56**/**0.91**/**0.98**
EEGNet-BMI-4	89.22/0.89/0.89	87.22/0.88/0.87	**91.94**/**0.91**/**0.91**	91.11/**0.91**/**0.91**
Shallow-BMI-4	91.91/0.91/0.91	90.92/0.91/0.90	92.57/**0.92**/**0.92**	**92.61**/**0.92**/**0.92**
Deep-BMI-4	63.80/0.73/0.63	87.22/0.88/0.87	**89.97**/**0.90**/**0.89**	89.05/0.89/**0.89**
ATCNet-BMI-4	84.65/0.84/0.92	77.82/0.77/0.85	84.88/0.84/0.91	**87.60**/**0.87**/**0.94**
Average	78.15/0.77/0.90	74.87/0.73/0.90	78.16/0.77/0.92	**81.84**/**0.81**/**0.93**

Some conclusions can be drawn from the above experimental results: (1) The fixed OFBCNN achieved or approached the highest performance using different setups and datasets. (2) The performance improvements provided by the fixed OFBCNN are universal for the CNN backbone, EEG dataset and low-cut frequency. (3) ATCNet with fixed OFBCNN achieved the highest accuracy in multisubject MI recognition tasks. (4) The performance of the nonoverlapping filter bank CNN was the worst because of the narrow bandpass filters applied before CNN.

**Fig. 2:**
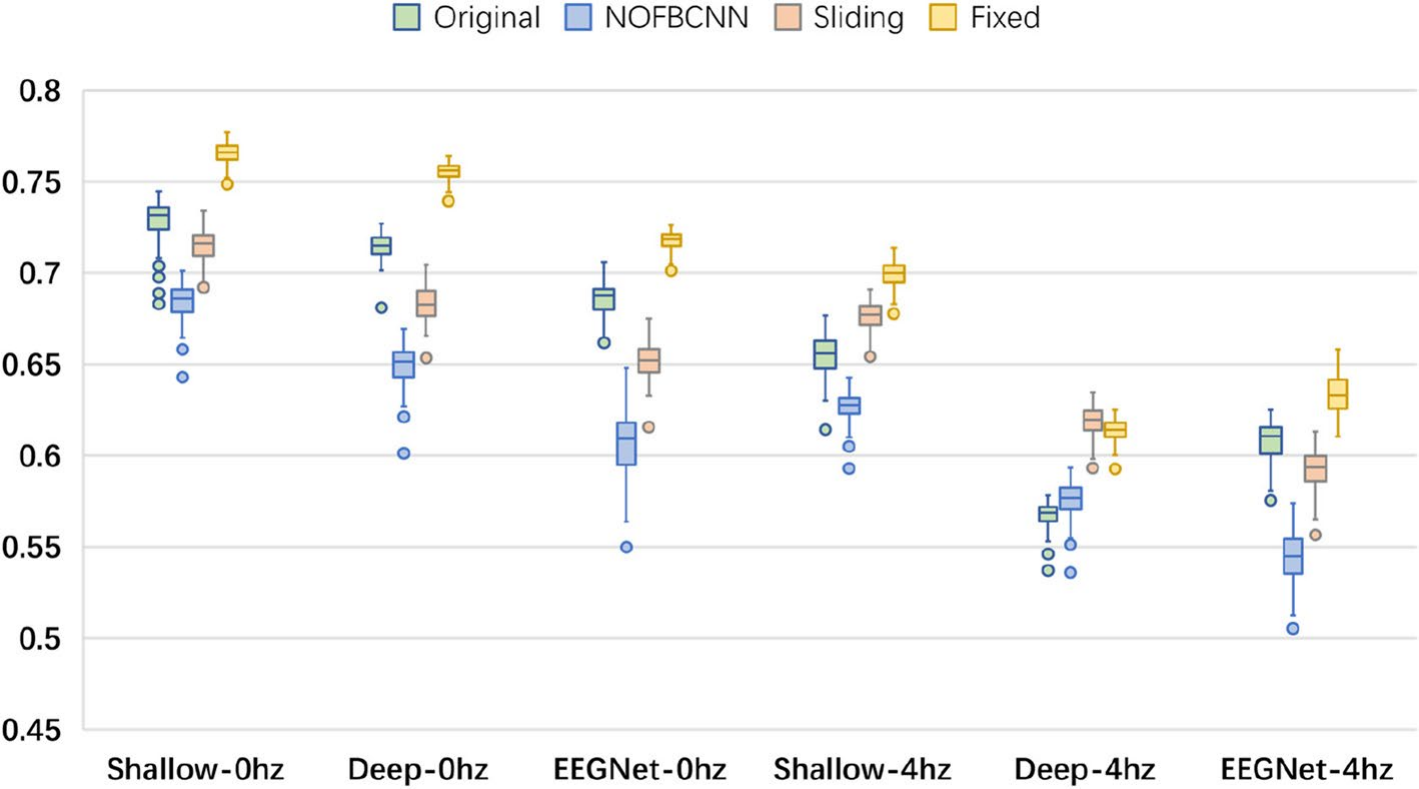
Accuracy boxplot comparison of fixed OFBCNN, sliding OFBCNN, NOFBCNN and original CNN for BCI Competition IV dataset 2a

Moreover, the accuracy boxplots of the fixed OFBCNN, sliding OFBCNN, NOFBCNN and original CNN for BCI Competition IV dataset 2a are compared in Fig. 2. The test accuracy was used to draw the boxplot. The upper and lower edges of the box represent the 25th and 75th percentiles of the accuracy, and the horizontal lines in the box represent the median of the data. The lines running above and below the box represent the maximum and minimum values. Outliers are marked with dots. As seen in the boxplot, the fixed OFBCNN achieved the highest or close to the highest accuracy in all configurations. In addition, the box body of the fixed OFBCNN was short, which indicates small fluctuations in accuracy. NOFBCNN had the lowest accuracy and the largest accuracy fluctuation due to insufficient discriminative information in narrow-band EEG signals.

**Fig. 3:**
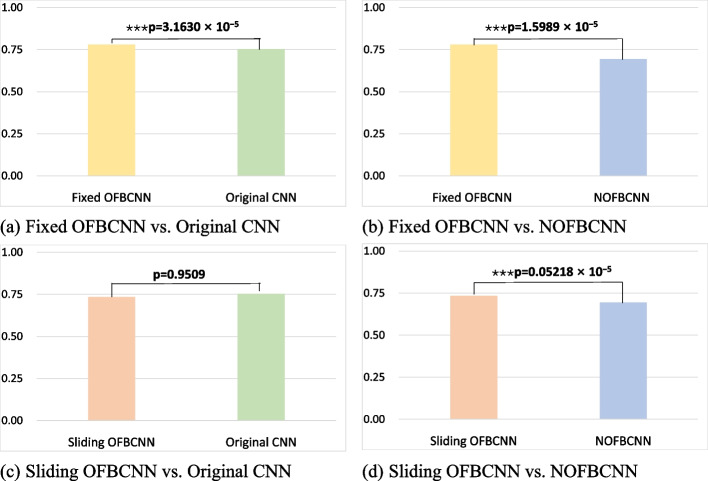
Evaluation of the statistical significance of the performance difference between the OFBCNN and original CNN or NOFBCNN. (*p < 0.05, **p < 0.01, ***p < 0.001)

To confirm the significance of the accuracy improvement, a paired-sample one-sided Student’s t test was conducted (the null hypothesis was that the accuracy of the OFBCNN model was equal to the accuracy of the original/NOFBCNN model, against the alternative that the accuracy of the OFBCNN model was greater than the accuracy of the original/NOFBCNN model). The p values are shown in Fig. 3. These results showed that the accuracy improvement of the fixed OFBCNN compared with the original model and NOFBCNN was significant.

To verify the convergence improvement provided by the OFBCNN, the variance in test accuracy in epochs 400–500 (epochs 200–300 in OpenBMI dataset) was compared with that of the original CNN and NOFBCNN by the variance in the test accuracy. The results are shown in Table 3. A smaller variance in the test accuracy means better convergence and stability.

**Table 3:** The convergence comparison (variance of accuracies (e-5)) of the proposed OFBCNN with the nonoverlapping filter bank CNN and original CNN in multisubject MI recognition

CNN-Dataset-Low-Cut Frequency	Original	NOFBCNN	Sliding OFBCNN	Fixed OFBCNN
EEGNet-2a-0 Hz	9.24	36.67	13.33	3.45
Shallow-2a-0 Hz	15.32	10.00	8.77	3.42
Deep-2a-0 Hz	12.11	16.67	10.00	3.53
EEGNet-2a-4 Hz	23.55	23.33	16.67	13.33
Shallow-2a-4 Hz	14.82	9.33	7.74	5.81
Deep-2a-4 Hz	10.85	13.15	8.67	5.00
EEGNet-HG-0 Hz	7.94	40.00	25.00	4.28
Shallow-HG-0 Hz	3.90	7.71	4.35	2.25
Deep-HG-0 Hz	13.33	85.00	35.00	3.88
EEGNet-HG-4 Hz	8.30	25.00	25.00	20.00
Shallow-HG-4 Hz	7.05	9.13	5.79	4.98
Deep-HG-4 Hz	60.00	105.00	45.00	20.00
Average	15.53	31.75	17.11	7.49

We can draw conclusions from Table 3 that the fixed OFBCNN was the most stable model, and the average improvement factor (the original CNN accuracy variance divided by the proposed fixed OFBCNN accuracy variance) was 2.06.

### Compared with the state-of-the-art algorithm

To fully show the superiority of the proposed method, the fixed OFBCNN was compared with the state-of-the-art algorithm on the BCI Competition IV dataset 2a. In addition to the backbone model, the following models were included in comparison:

- CNN++ [28]: CNN++ is an improved version of the CNN model consisting of 5 CNN and max pooling layers with an input fully connected (FC) layer. Inspired by CSP, a channel projection by a fully connected layer is conducted before the convolution layer.
- PSTNet [29]: PSTNet is a CNN architecture based on a self-attention mechanism that extracts distinguishable spatial-temporal features through the attention mechanism in the time domain and space domain.
- TS_SEFFNet [14]: TS_SEFFNet designed the DT-Conv block and MS-Conv block for feature extraction and finally used the SE-Feature-Fusion block for feature fusion.

**Fig. 4:**
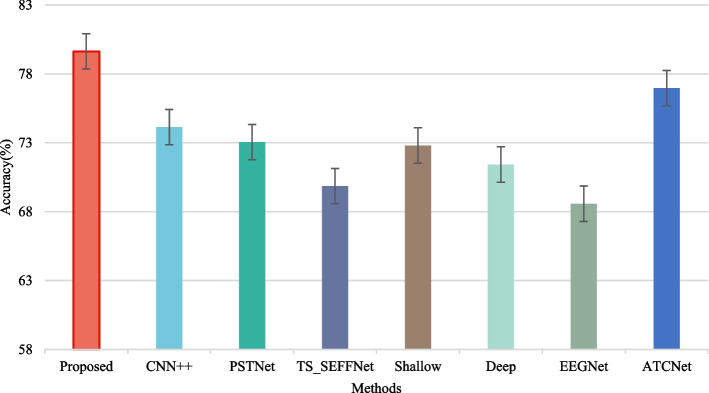
Performance comparison with state-of-the-art algorithms

The maximum number of epochs for training was also set as 500 for performance comparison, and other configurations are the same as those in the original literature. The average test accuracy was used for comparison, and the results are shown in Fig. 4. The proposed method showed a higher accuracy than the other state-of-the-art algorithms in the comparison.

### Parameter selection

**Fig. 5:**
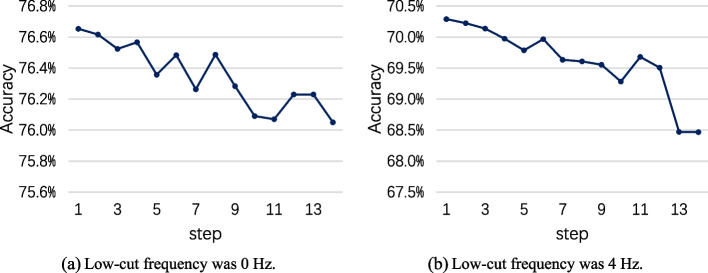
The average testing accuracy comparison of different frequency steps

The key parameter in the proposed OFBCNN framework is the frequency step of the filter bank. In previous results, the frequency step of bandpass filters was set as 4 Hz. The influence of different frequency steps was tested in this section. As the excellent capability of the fixed OFBCNN based on Shallow ConvNet was shown previously, it was employed in the test on the BCI Competition IV dataset 2a, and the results are shown in Table 4. The first bandpass filter was set as shown in Table 1, and the following bandpass filters were set according to the frequency step. For example, if the low-cut frequency was 0 Hz and the frequency step was 1 Hz, the bandpass filter frequency of the overlapping filter bank was 0–8 Hz, 0–9 Hz, 0–10 Hz, …, 0–36 Hz, and thus, this overlapping filter bank had 29 bandpass filters. To visually show the influence of the frequency step on the recognition accuracy, the results in Table 4 are shown in Fig. 5.

**Table 4:** The average testing accuracy comparison of different frequency steps

Step	1 Hz	2 Hz	3 Hz	4 Hz	5 Hz	6 Hz	7 Hz	8 Hz	9 Hz	10 Hz	11 Hz	12 Hz	13 Hz	14 Hz
0 Hz	76.65	76.62	76.52	76.57	76.36	76.48	76.26	76.49	76.28	76.09	76.07	76.23	76.23	76.05
4 Hz	70.29	70.22	70.14	69.97	69.79	69.97	69.63	69.61	69.55	69.28	69.68	69.51	68.47	68.47

If the frequency step is smaller, more bandpass filters will exist in the OFBCNN, and more CNN models need to be trained. In contrast, if the frequency step is larger, fewer bandpass filters will exist. However, the OFBCNN cannot take advantage of the discriminative information from all frequency bands, and the probability ensemble of a few models will diminish the discriminatory ability. Therefore, the selection of the frequency step in the filter bank was a compromise between performance and computational complexity. Based on the results in Table 4, 4 Hz was selected as an empirical value.

## Conclusion

In this paper, an overlapping filter bank CNN framework was proposed to enable the CNN model to learn discriminative information from multiple frequency bands of EEG for multisubject MI-BCI. Specifically, the novel overlapping filter bank with a fixed low cut frequency and overlapping filter bank with a sliding low cut frequency were applied for preprocessing EEG. Comprehensive experimental evaluations using two benchmark datasets were conducted to test the effectiveness and universality of the proposed overlapping filter bank CNN framework. The experimental results showed that the fixed OFBCNN achieved the highest classification accuracy.

## Data Availability

The datasets used and/or analysed during the current study are from public datasets.
